# Supplementary material for: Niche availability and competitive loss by facilitation control proliferation of bacterial strains intended for soil microbiome interventions
Source: Nat Commun. 2024 Mar 22;15:2557. doi: 10.1038/s41467-024-46933-1 (PMC10959995; doi:10.1038/s41467-024-46933-1)
Supplement: Supplementary file 1 — Supplementary Information [file 41467_2024_46933_MOESM1_ESM.pdf]

SUPPLEMENTARY INFORMATION

Niche Availability and Competitive Loss by Facilitation Control Proliferation of Bacterial Strains Intended for Soil Microbiome Interventions

Senka Čaušević, Manupriyam Dubey, Marian Morales, Guillem Salazar, Vladimir Sentchilo, Nicolas Carraro, Hans-Joachim Ruscheweyh, Shinichi Sunagawa, and Jan Roelof van der Meer

INVENTORY OF SUPPLEMENTARY INFORMATION

Supplementary Figures 1-9
Supplementary Tables 1

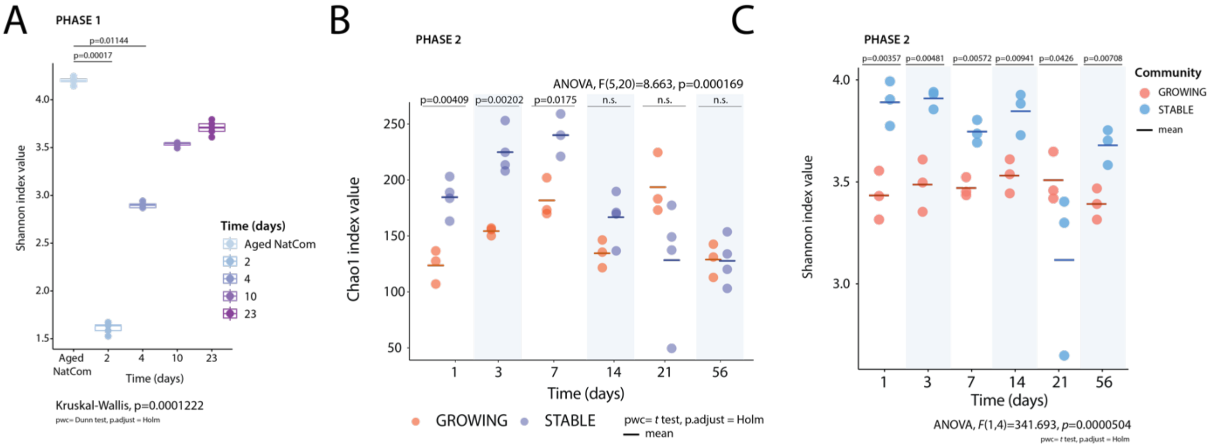

Supplementary Fig. 1. Changes in diversity indices of Phase 1 and Phase 2 NatComs. (A) Shannon diversity measures calculated at amplicon sequence variant (ASV) level over time (shades from blue to magenta) for revived NatComs in Phase 1 and the aged NatCom inoculum. Dots represent individual values of five replicates. *P*-values are indicated if <0.05 after Holm's *p*-value adjustment. **(B)** and **(C)** Chao1 and Shannon diversity values for Phase 2 GROWING (orange dots) and STABLE (blue dots) NatComs over time. Horizontal lines indicate mean values of biological replicates. *P*-values above the plot refer to pairwise comparisons (t-test on ranked values, Holm's adjustment). ANOVA refers to a repeated measures ranked two-way ANOVA, reporting the significant effect of NatCom growth phase.

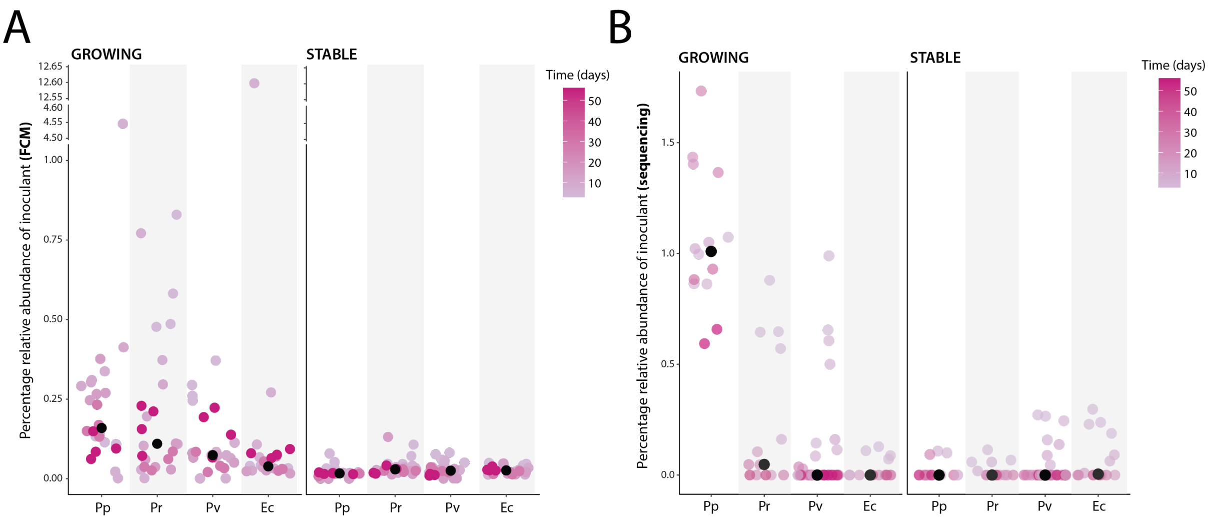

Supplementary Fig. 2. Relative abundances of inoculants in Phase 2 GROWING or STABLE NatComs. (A) Relative abundance of each inoculant over time (time represented by the shade of magenta, according to the indicated scale), calculated by dividing the flow cytometry (FCM) counts of the inoculant (based on their mCherry fluorescent signal) by the corresponding total community counts (Syto9-stained signal) and expressed as percentage. Black dots represent the median of pooled replicates and time points per inoculant. **(B)** As in (A), with relative abundances calculated from community 16S rRNA gene amplicon composition data.

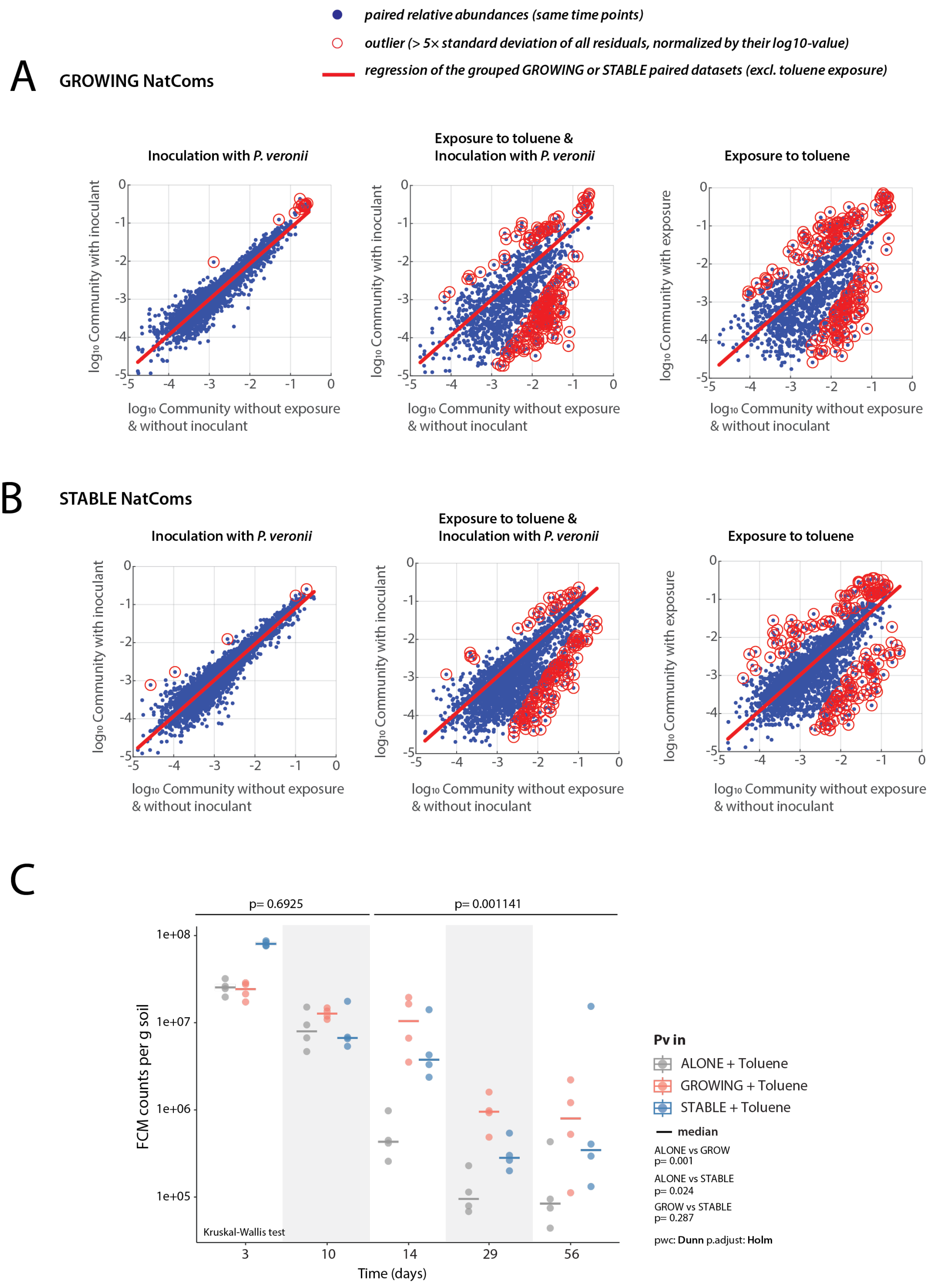
paired relative abundances (same time points)
outlier (> 5× standard deviation of all residuals, normalized by their log10-value)
regression of the grouped GROWING or STABLE paired datasets (excl. toluene exposure)
A
GROWING NatComs
Inoculation with P. veronii
Exposure to toluene & Inoculation with P. veronii
Exposure to toluene
log10 Community with inoculant
log10 Community with exposure
log10 Community without exposure & without inoculant
B
STABLE NatComs
C
p= 0.6925
p= 0.001141
FCM counts per g soil
Kruskal-Wallis test
Time (days)
Pv in
ALONE + Toluene
GROWING + Toluene
STABLE + Toluene
median
ALONE vs GROW p= 0.001
ALONE vs STABLE p= 0.024
GROW vs STABLE p= 0.287
pwc: Dunn p.adjust: Holm

Supplementary Fig. 3. Effect of toluene addition on NatCom state and inoculant persistence. (A) Paired ASV abundances in GROWING NatComs exposed or not to toluene, and with or without inoculated *P. veronii*. Dots represent time-paired $\log_{10}$ transformed relative abundances of the same amplicon sequence variant (ASV) across all biological replicates (arbitrarily paired among replicates) and treatments, as indicated. Dots with read circles highlight ASVs considered enriched or depleted compared to the linear regression model of all GROWING data sets (excluding toluene exposure itself). Threshold for being an outlier is taken as having an y-residual to the regression line more than five times the standard deviation across all regression residuals, corrected for their $\log_{10}$ value. Note the dramatic effect on the appearance of outliers upon exposure to toluene. **(B)** As (A) but for the STABLE NatComs. For a listing of all outlier taxa names, see Table S2. **(C)** *P. veronii* (Pv) population sizes in microcosms exposed to toluene either ALONE or in presence of GROWING or STABLE resident community. Lines indicate median value of biological replicates. *P*-values above plots correspond to Kruskal-Wallis comparisons, grouped per 'early' (Day 3 and 10) and 'late' time points (Day 14, 29, and 56). *P*-values on the side correspond to Dunn pairwise comparisons for the late phase sizes of the *P. veronii* populations.

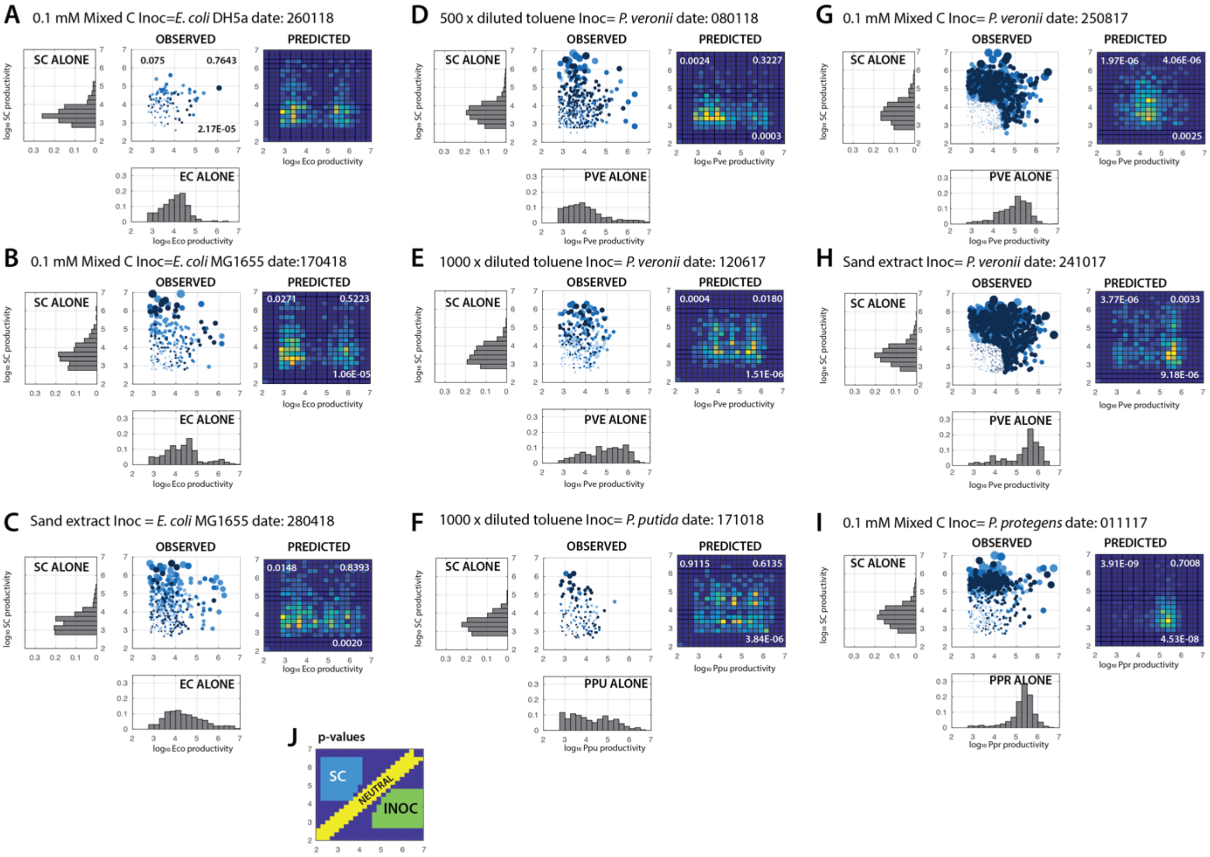

Supplementary Fig. 4. Expected versus observed paired productivities of inoculants with random resident soil cells (SC). (A) – (I) Paired productivity plot of beads with only a single inoculant and a single soil cell microcolony (OBSERVED) versus beads with inoculant or soil cells alone (summed from time points 24, 48, and 72 h). Circles are proportional to the sum of the measured microcolony sizes (light to dark blue represent time points 0, 6, 24, 48, and 72 h). The heatmap (PREDICTED) shows the expected paired bead summed productivities from the individual measured microcolony sizes (i.e., inoculant and SC ALONE) for the same number of beads as analyzed by microscopy, with yellow colors corresponding to high incidence and dark blue to low. *P*-values correspond to the two-tailed t-test comparison of the variation of the total measured paired productivities inside the three regions (n = 3; 24, 48, and 72 h) to that in the simulations (n = 5), as indicated in panel J. The upper left region shows higher SC productivity than expected, the lower right regionshows lower inoculant productivity than expected, and the diagonal shows thesame productivity for both microcolonies in a pair (EC =*E. coli*, PVE =*P. veronii*, PPU = *P. putida*, and PPR = *P. protegens*).

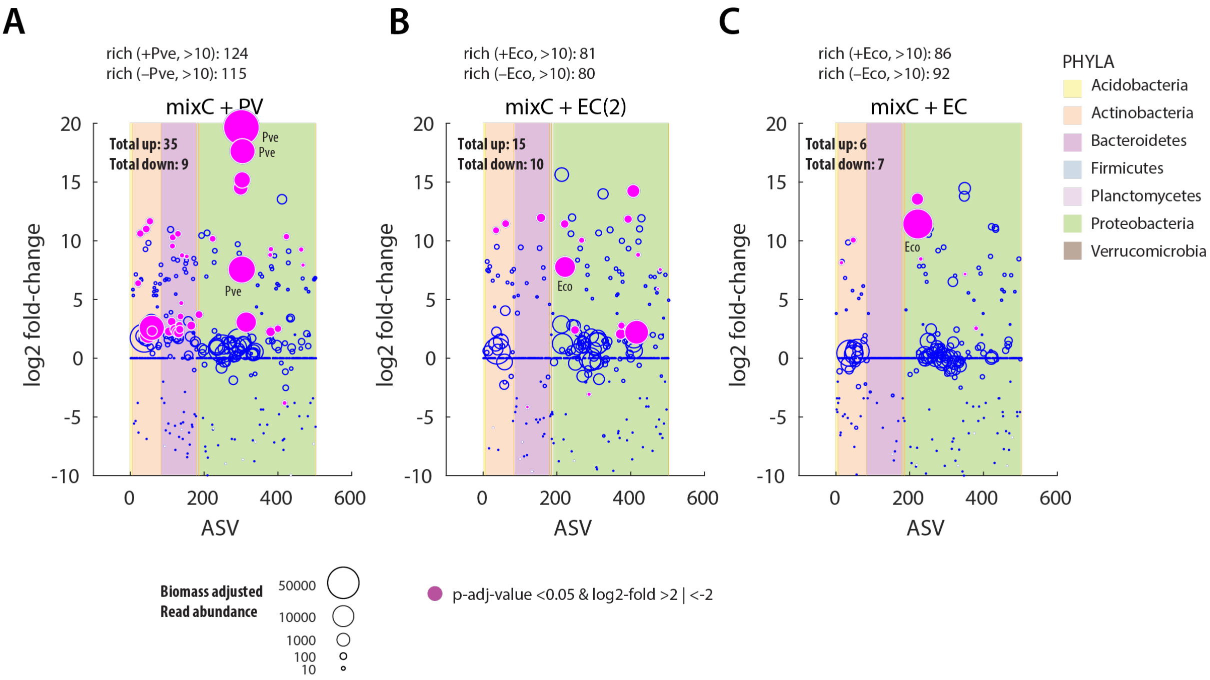

Supplementary Fig. 5. Effect of *P. veronii* or *E. coli* on taxa enrichment or depletion in paired agarose bead incubations with mixed C as substrates. (A)-(C) Mean $\log_2$ -fold change of absolute read abundances of individual ASVs (corrected for imaged biomass differences; only ASVs with more than 10 reads and inoculant reads retained) in bead-encapsulated communities with or without *P. veronii* (A) or *E. coli* (B and C, two independent incubation series), and incubated with mixed carbon substrates after 48 h. Background colors show phyla attribution. Dots indicate mean values from biological triplicates with a circle size proportional to the absolute read abundance in the data set. Rich, ASV richness. Magenta dots denote significantly different taxa abundance changes ($\log_2$-fold >2 or <–2; adjusted P-value < 0.05). Eco and Pse, indication of the inoculants themselves.

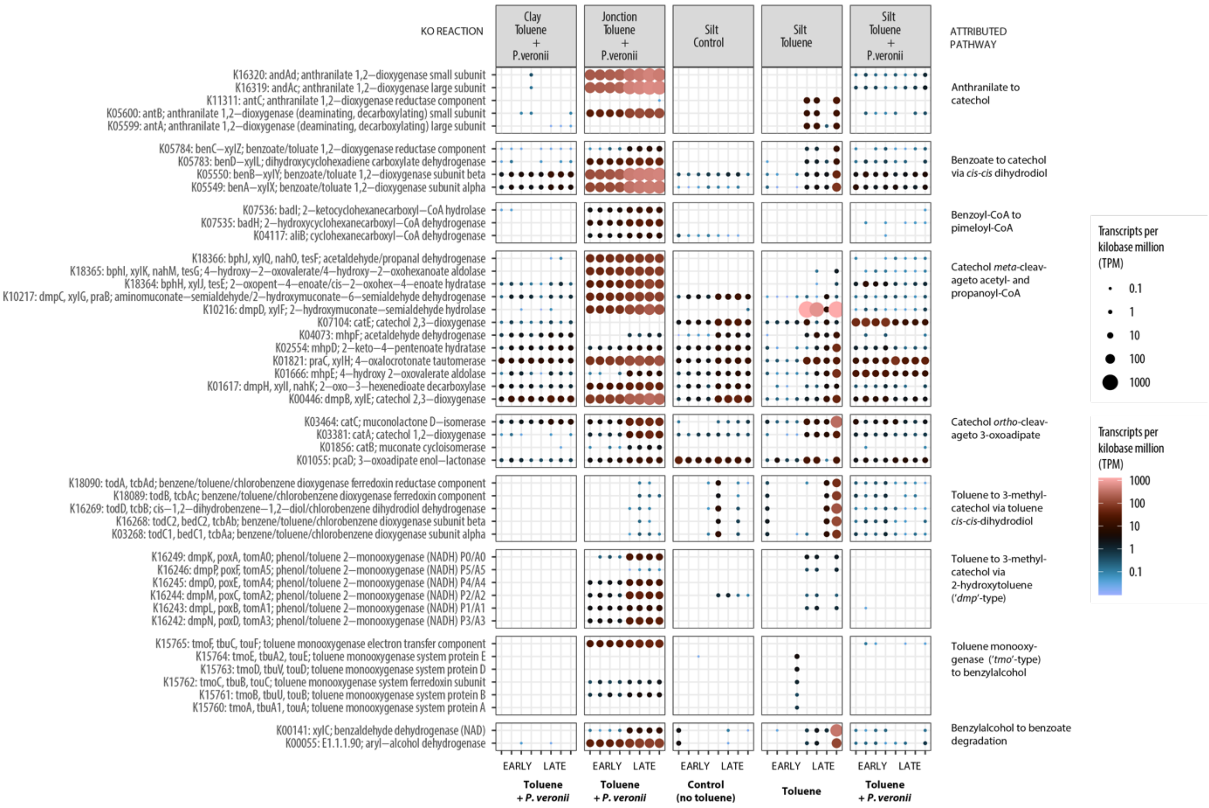

Supplementary Fig. 6. Enrichment of toluene degradation pathways in soil microbiota in presence or absence of added *P. veronii*. Plots show assigned transcript abundances (in TPM (transcripts per kilobase million) as circle sizes and colors according to scales on the right) in the various sample incubations for KEGG orthology reactions (as specified on the left of the panels and grouped per pathway on the right). TPM values of transcripts annotated to the aromatic compound metabolic steps on the right for the different soils, conditions, and timepoint (early or late, see panel A of Fig. 6 in the main text). Data points show individual values from quadruplicate experiments.

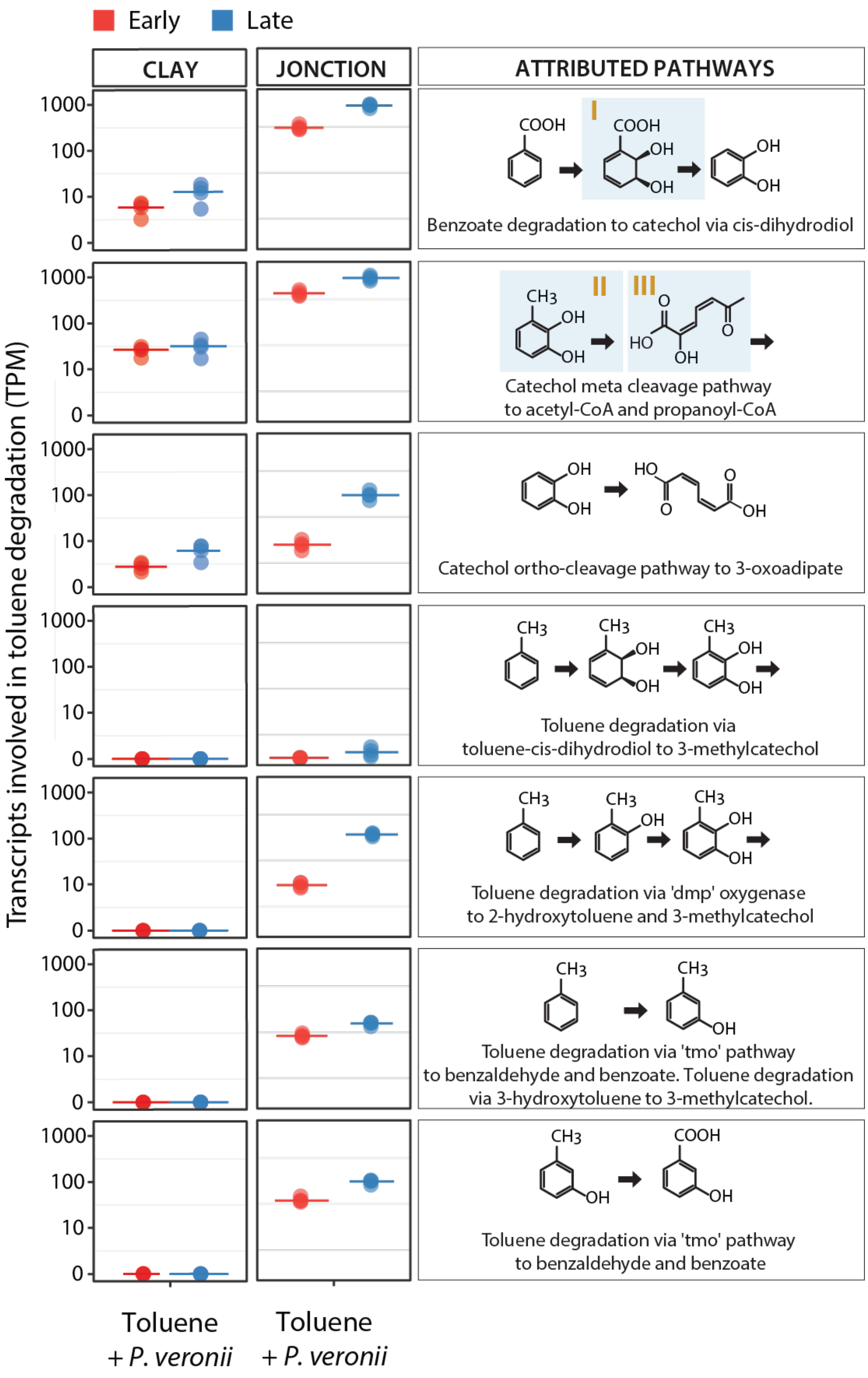

Supplementary Fig. 7. Exploitation of toluene degradation products from *P. veronii* by microbiota in Clay and Jonction. TPM values of transcripts annotated to the aromatic compound metabolic steps on the right for Clay and Jonction soils inoculated with *P. veronii* and tested at two timepoints (early or late, Fig. 6A). Data points show individual values from quadruplicate experiments, presented as dots, and a line indicating their median value.

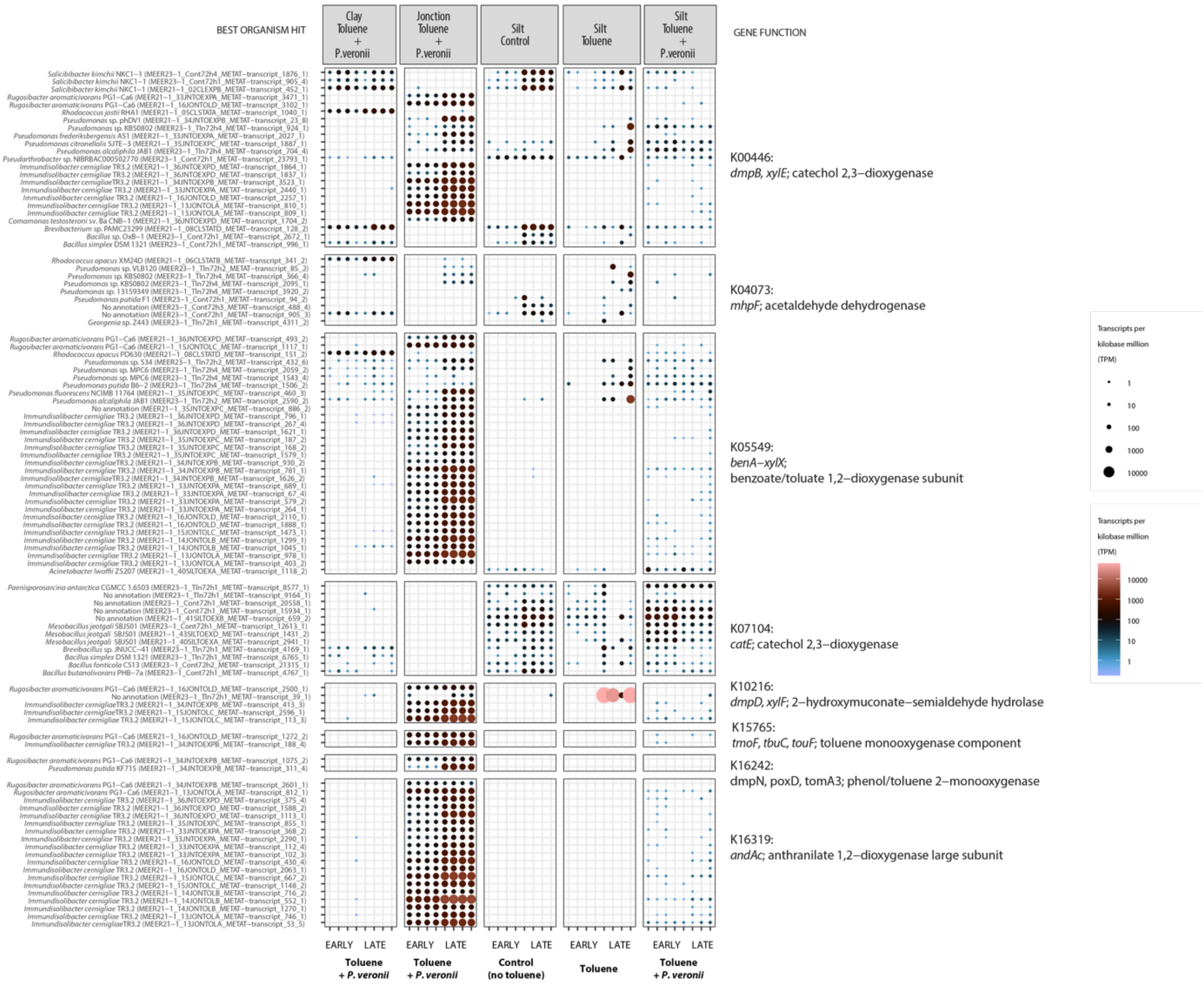

Supplementary Fig. 8. Taxa attribution of enriched toluene degradation pathways among soil microbiota in the presence or absence of added *P. veronii*. Plots show assigned transcript abundances (in TPM as circle sizes and colors according to scales on the right) for specific KEGG orthology reactions and representative genes (as specified on the right of the panels) attributed to the best-hit taxa score (on the left). TPM values of transcripts for the different soils, conditions, and timepoints (early or late, see panel A of Fig. 6 in the main text). Data points show individual values from quadruplicate experiments.

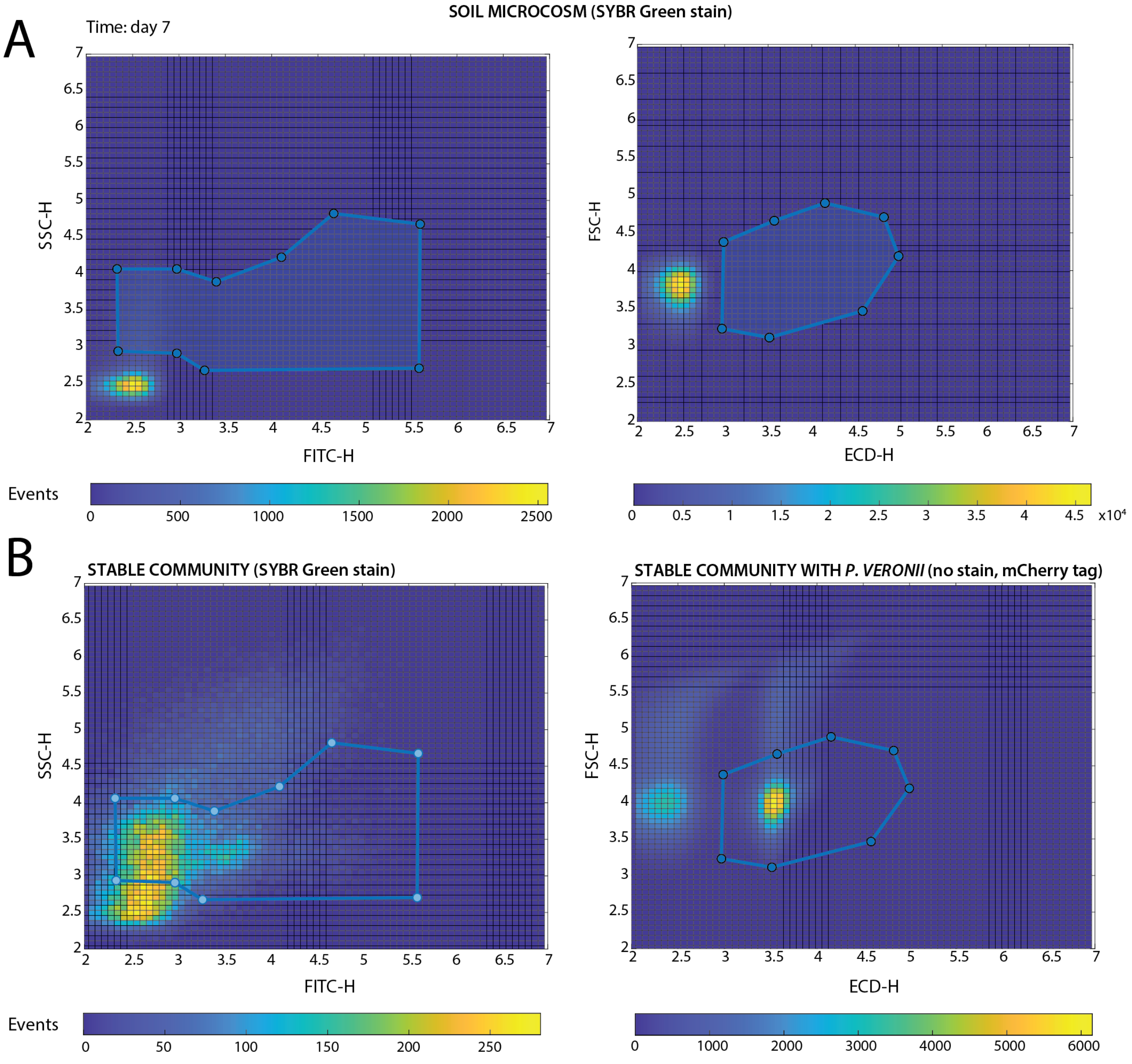

Fig. S9. Gating procedure for microcosm community and inoculant population size measurement. (A) SSC-H *vs*. FITC-H and FSC-H *vs*. ECD-H profile of soil sample taken at day 7 from uninoculated sterile microcosm (negative control) stained with SYBR Green I. Polygon on the left indicates gate coordinates for the total community, and on the right for inoculant events. **(B)** As in (A), example of SYBR Green I stained stable community sample (on the left) and unstained stable community with *P. veronii* sample (on the right). Note the change in gate content in comparison to respective negative controls in (A).

Supplementary Table S1: Composition of 16 carbon source minimal medium for soil bacteria growth.

	Compound name	Mw	No C-atoms	Equivalent mM C	Conversion for nr of C-atoms	Mass to weigh (mg per L)
1	L-Arginine	174.2	6	0.451	0.07518797	13.10
2	D-Xylose	150.1	6	0.451	0.07518797	11.29
3	L-Aspartic acid potassium salt	209.3	4	0.301	0.07518797	15.74
4	4-Hydroxybenzoic acid	144.0	7	0.526	0.07518797	10.83
5	L-Serine	105.1	3	0.226	0.07518797	7.90
6	beta-Hydroxy Butyric Acid	104.1	4	0.301	0.07518797	7.83
7	D-Cellobiose	342.3	12	0.902	0.07518797	25.74
8	alpha-D-Lactose	360.3	12	0.902	0.07518797	27.09
9	Putrescine	88.15	4	0.301	0.07518797	6.63
10	Itaconic acid	130.1	5	0.376	0.07518797	9.78
11	Alpha-D-glucose-1-phosphate	304.1	6	0.451	0.07518797	22.86
12	N-acetyl-D-glucosamine	221.2	8	0.602	0.07518797	16.63
13	D-Mannitol	182.2	6	0.451	0.07518797	13.70
14	Meso-erythitol	112.2	4	0.301	0.07518797	8.44
15	Galacturonic acid	194.1	6	0.451	0.07518797	14.59
16	Tween 20	604.8	40	3.008	0.07518797	45.47
	Total		133 C	10		

Final concentration for C = 0.1 mM
Equivalent for 10 mM C on proportion of C-atoms in the compound (e.g., 6/133*10=0.451)
Conversion factor, e.g., 0.451/6=0.07519 mM of the compound to be added
Mass to weigh: conversion factor x Mw, e.g., 0.07519 * 174.2 = 13.10 mg per L
