# Supplementary material for: Niche availability and competitive loss by facilitation control proliferation of bacterial strains intended for soil microbiome interventions
Source: Nat Commun. 2024 Mar 22;15:2557. doi: 10.1038/s41467-024-46933-1 (PMC10959995; doi:10.1038/s41467-024-46933-1)
Supplement: Supplementary file 3 — Description of Additional Supplementary Files [file 41467_2024_46933_MOESM3_ESM.pdf]

Description of Additional Supplementary Files

File Name: Supplementary Data 1
Description: Statistical procedures and test results for each figure panel (separate sheet in the Excel file).

File Name: Supplementary Data 2
Description: Outlier taxa in inoculated compared to non-inoculated soil microcosms. See also Fig. 3c.

File Name: Supplementary Data 3
Description: Outlier taxa in inoculated compared to non-inoculated soil microcosms in presence of toluene. See also Fig. 3d and Supplementary Fig. 3.

File Name: Supplementary Data 4
Description: Diversity measures of bead communities after 48 h incubation. See also Fig. 5.

File Name: Supplementary Data 5
Description: Taxa outliers in inoculant-soil cell paired bead incubations. See also Fig. 5b.

File Name: Supplementary Data 6
Description: KEGG identifiers in toluene or toluene intermediate degradation. See also Fig. 6c.
