# Supplementary material for: Niche availability and competitive loss by facilitation control proliferation of bacterial strains intended for soil microbiome interventions
Source: Nat Commun. 2024 Mar 22;15:2557. doi: 10.1038/s41467-024-46933-1 (PMC10959995; doi:10.1038/s41467-024-46933-1)
Supplement: Supplementary file 10 — Reporting Summary [file 41467_2024_46933_MOESM10_ESM.pdf]

nature portfolio

Corresponding author(s): van der Meer

Last updated by author(s): March 4, 2024

Reporting Summary

Nature Portfolio wishes to improve the reproducibility of the work that we publish. This form provides structure for consistency and transparency in reporting. For further information on Nature Portfolio policies, see our Editorial Policies and the Editorial Policy Checklist.

Statistics

For all statistical analyses, confirm that the following items are present in the figure legend, table legend, main text, or Methods section.

n/a	Confirmed	
☐	☒	The exact sample size (n) for each experimental group/condition, given as a discrete number and unit of measurement
☐	☒	A statement on whether measurements were taken from distinct samples or whether the same sample was measured repeatedly
☐	☒	The statistical test(s) used AND whether they are one- or two-sided<br>*Only common tests should be described solely by name; describe more complex techniques in the Methods section.*
☐	☒	A description of all covariates tested
☐	☒	A description of any assumptions or corrections, such as tests of normality and adjustment for multiple comparisons
☐	☒	A full description of the statistical parameters including central tendency (e.g. means) or other basic estimates (e.g. regression coefficient) AND variation (e.g. standard deviation) or associated estimates of uncertainty (e.g. confidence intervals)
☐	☒	For null hypothesis testing, the test statistic (e.g. F, t, r) with confidence intervals, effect sizes, degrees of freedom and P value noted<br>*Give P values as exact values whenever suitable.*
☒	☐	For Bayesian analysis, information on the choice of priors and Markov chain Monte Carlo settings
☐	☒	For hierarchical and complex designs, identification of the appropriate level for tests and full reporting of outcomes
☒	☐	Estimates of effect sizes (e.g. Cohen's d, Pearson's r), indicating how they were calculated

Our web collection on statistics for biologists contains articles on many of the points above.

Software and code

Policy information about availability of computer code

Data collection	Custom MATLAB (v. 2021b) image processing routine that segments agarose beads and microcolonies inside beads. Reference 62 (http://doi.org/10.5281/zenodo.4568347).<br>CytoFLEX Flow Cytometer (Beckman Coulter) instrument software to collect stained bacteria events for counting community and population sizes.

Data analysis

Raw sequence reads of 16S rRNA gene amplicons were analyzed using the Qiime2 platform on UNIX (version 2021.8), and amplified sequence variants (ASVs) were attributed to known taxa at 99% identity (operational taxonomic units, OTU) by comparison to the SILVA database (version 132).
Flow cytometry data was imported using the function fca_readfcs and analyzed using custom MATLAB scripts (v. 2021b).
Microcolony growth was quantified using custom MATLAB (v. 2021b) scripts.
Metatranscriptomic sequencing reads from all samples were quality controlled by BBMap (v.38.71). The reads from metatranscriptomic samples were assembled into transcripts using the SPAdes assembler (v3.15.2) in transcriptome mode. Gene sequences were predicted using Prodigal (v2.6.3). Gene sequences from the GenBank entry of P. veronii (GCA_900092355) were downloaded and clustered at 95% identity, keeping the longest sequence as representative using CD-HIT (v4.8.1). Representative gene sequences were aligned against the KEGG database (release April.2022) using DIAMOND 55 (v2.0.15).
Data processing, analysis of community composition, and statistical analysis were done using GraphPad Prism (version 9.0.1) and R 4.0 (R Core Team, 2019) on RStudio (version 2022.2.3.492) using the following packages: phyloseq, microbiome, MicrobiotaProcess, ggplot2, vegan, biomformat, tidyverse, reshape2, Biostrings, PMCMRplus, emmeans, and RVAideMemoire.

The 145 metatranscriptome samples were mapped to the 246,873cluster representatives with BWA (v0.7.17-r1188; -a).

For manuscripts utilizing custom algorithms or software that are central to the research but not yet described in published literature, software must be made available to editors and reviewers. We strongly encourage code deposition in a community repository (e.g. GitHub). See the Nature Portfolio guidelines for submitting code & software for further information.

Data

Policy information about availability of data

All manuscripts must include a data availability statement. This statement should provide the following information, where applicable:

- Accession codes, unique identifiers, or web links for publicly available datasets
- A description of any restrictions on data availability
- For clinical datasets or third party data, please ensure that the statement adheres to our policy

Raw metatranscriptomic datasets of P. veronii inoculation into Clay, Silt, and Jonction are available from Bioproject accession number PRJNA682712 [https://www.ncbi.nlm.nih.gov/bioproject/?term=PRJNA682712], and datasets depleted from P. veronii reads itself can be accessed from the European Nucleotide Archive (accession numbers,
ERS2210331 [https://www.ebi.ac.uk/ena/browser/view/ERS2210331],
ERS2210332 [https://www.ebi.ac.uk/ena/browser/view/ERS2210332],
ERS2210333 [https://www.ebi.ac.uk/ena/browser/view/ERS2210333],
ERS2210334 [https://www.ebi.ac.uk/ena/browser/view/ERS2210334],
ERS2210335 [https://www.ebi.ac.uk/ena/browser/view/ERS2210335],
ERS2210336 [https://www.ebi.ac.uk/ena/browser/view/ERS2210336],
ERS2210337 [https://www.ebi.ac.uk/ena/browser/view/ERS2210337],
ERS2210338 [https://www.ebi.ac.uk/ena/browser/view/ERS2210338],
ERS2210339 [https://www.ebi.ac.uk/ena/browser/view/ERS2210339],
ERS2210340 [https://www.ebi.ac.uk/ena/browser/view/ERS2210340],
ERS2210341 [https://www.ebi.ac.uk/ena/browser/view/ERS2210341],
ERS2210342 [https://www.ebi.ac.uk/ena/browser/view/ERS2210342],
ERS2210343 [https://www.ebi.ac.uk/ena/browser/view/ERS2210343],
ERS2210344 [https://www.ebi.ac.uk/ena/browser/view/ERS2210344],
ERS2210345 [https://www.ebi.ac.uk/ena/browser/view/ERS2210345],
ERS2210346 [https://www.ebi.ac.uk/ena/browser/view/ERS2210346]). The raw 16S rRNA gene V3-V4 amplicon sequences for the random-paired inoculant-soil taxa bead communities incubated under different substrate conditions can be accessed from the Short Read Archives under BioProject ID PRJNA661487 [https://www.ncbi.nlm.nih.gov/sra/?term=PRJNA661487]. Finally, NatCom community profiling by 16S rRNA gene amplicon analysis is accessible through BioProject ID PRJNA1024897 [https://www.ncbi.nlm.nih.gov/bioproject/?term=PRJNA1024897].

A single downloadable ZIP-file with raw data, processed data, numerical values underlying all Figure and supplementary figure parts, and including all the R and MATLAB scripts used, has been uploaded to Zenodo (Ref. 88). https://doi.org/10.5281/zenodo.10517751.

All genes from bacterial and archaeal genomes annotated to the corresponding KEGG orthologs (K15765, K16242, K00446, K07104, K04073, K10216, K05549, K16319) in IMG/M (Integrated microbial genomes and microbiomes: https://img.jgi.doe.gov/) were downloaded and used as a reference database to annotate all genes from the metatranscriptomics data with the same KEGG ortholog assignment.

Representative gene sequences were aligned against the KEGG database (release April 2022; https://www.genome.jp/kegg/).

Research involving human participants, their data, or biological material

Policy information about studies with human participants or human data. See also policy information about sex, gender (identity/presentation), and sexual orientation and race, ethnicity and racism.

Reporting on sex and gender	N/A
Reporting on race, ethnicity, or other socially relevant groupings	N/A

Population characteristics	N/A
Recruitment	N/A
Ethics oversight	N/A

Note that full information on the approval of the study protocol must also be provided in the manuscript.

Field-specific reporting

Please select the one below that is the best fit for your research. If you are not sure, read the appropriate sections before making your selection.

☐ Life sciences ☐ Behavioural & social sciences ☒ Ecological, evolutionary & environmental sciences

For a reference copy of the document with all sections, see nature.com/documents/nr-reporting-summary-flat.pdf

Ecological, evolutionary & environmental sciences study design

All studies must disclose on these points even when the disclosure is negative.

Study description

For the first experimental setup with standardized soil communities we produced and used fifty (50) replicates of soil microcosm flasks to culture soil communities. Five of those were randomly selected and sampled during 28 days to follow community growth and for compositional analysis. The others were incubated in parallel but not sampled.
After 28 days of growth all inoculated microcosms were pooled, mixed and divided into two new sets of 28 microcosms. One set (called STABLE microcosms) was used directly for inoculations or controls. For the other set (called GROWING) the soil was mixed one-to-ten with fresh microcosm material to allow new growth and simultaneously inoculated or not.
For each set of microcosm experiments, we then had four replicates of each inoculant (of which there were four), an inoculated microcosm in 4 replicates, a set of 4 replicates receiving toluene dosage, and a set of four with one specific inoculant (Pseudomonas veronii) and toluene.

For the second experimental setup with random paired growth experiments between inoculants and soil bacteria, we used triplicate biological replicates with always encapsulated soil bacteria alone, inoculant alone and encapsulated soil bacteria paired with inoculant. Then we tested four different inoculants with three substrate conditions (but not in all possible combinations). For every experiment, we freshly isolated the soil bacteria from their natural habitat. With Pseudomonas veronii we did two independently started conditions of mixed-carbon substrates, one experiment with soil extract and two with toluene. For Escherichia coli we did two independently started experiments with mixed-carbon substrates, and one with sand extract. For Pseudomonas putida we used toluene. For Pseudomonas protegenes we used mixed carbon substrate conditions.

For the third experimental setup of the metatranscriptomics we used three soils (labeled as a 'clay', 'silt' and 'Jonction contaminated material'), which were inoculated with Pseudomonas veronii and supplemented with toluene. For one type (the 'silt'), we performed control incubations without inoculant (but with toluene) or without inoculant and without toluene. These microcosm incubations were conducted in four replicates each.

Research sample

For the first experimental setup with standardized soil communities we sampled 10 g soil at each time point, foreseeing timepoints after 3, 7, 10, 14 21, 29 and 56 days. The soil microcosm was placed on a bottle roller before sampling and sterile single-use spatula were used for the sampling. Microbial cells were extracted from the soil, then concentrated, after which total DNA was isolated for the 16S rRNA gene amplicon sequencing and community characterization. The same extracted cell suspension was used to quantify the number of cells by flow cytometry and colony forming units.

For the second experimental setup with random paired growth experiments we sampled the agarose bead suspensions at start and then at 6, 24, 48 and 72 h of growth. A volume of 10 µl of bead suspension was removed from the vials for each replicate. We imaged between 10 and 20 areas, to cover between 500 and 1000 beads with cells.

For the third experimental setup of the metatranscriptomics we again sampled the soils at two time points that we aimed were corresponding to the exponential growth phase of Pseudomonas veronii in the soil, and its stationary phase. 10 gram material was removed from each replicate microcosms, from which the cells were washed and recovered, before using for RNA isolation. We use here the raw metatranscriptomics sequences that were obtained from a previous experiment in which we had only focused on the gene expression of Pseudomonas veronii itself.

Sampling strategy

Sample sizes were essentially driven by the amount of DNA or RNA that we aimed to recover for subsequent library preparation for sequencing. The agarose bead sample size was determined by the volume we could place on a microscope slide and have proper individual bead imaging.

Data collection

For the standardized soil communities we collected data after 3, 7, 10, 14, 21, 29 and 56 days - and in one repetition of the experiment, at start, 10h, 24h and 32 h. As mentioned, samples here were used to determine community growth, inoculant growth and community composition. Data were recorded by S. C.
For the agarose bead experiments we again sampled with a logic to follow community growth - hence at 6h, 24h, 48 and 72 h. Here we focused on imaging and recording growth into microcolonies, which would be indicative for paired interactions. Data were recorded by M. D.
For the metatranscriptomic experiments we aimed at two time points of 'exponential growth' for the inoculant in the soil and a stationary phase of its growth, in order to capture the possible reactions of resident bacteria. These data were recorded by M. M.

Timing and spatial scale	See Data collection above. No relevant spatial scale was included here.
Data exclusions	Some bead experiments were excluded because of bad bead preparations (broken beads) and lots of visible growth outside beads.
Reproducibility	Standardized soil microcosm experiments were run in four replicates, ensuring their reproducibility. No data or replicates were discarded.<br>Agarose bead experiments were started with independently washed soil bacteria from fresh material, and independent repetitions with the same substrate conditions were started. This compromises somewhat the direct reproducibility but we argued that having fresh soil material was more important to ensure as much as possible the viability and composition of the natural material (instead of e.g., freezing a large sample at –80°C). All independent replicates were successful.<br>The metatranscriptomic experiments were run in four replicates but all started at the same time, except in one case of the 'silt'; where the uninoculated control was carried out at a later time point using the same source of fresh material (these soils were not stored but directly sampled). All independent replicates were successful. Unfortunately, we did not have sufficient material of the Jonction contaminated site to conduct uninoculated control experiments, because this site (in Geneva) was remediated and closed.
Randomization	Soil microcosms were randomized into the various groups. Bead experiments and microcosms for metatranscriptomic studies were not randomized because of the small smaple size.
Blinding	Data were not acquired blindly, because we would not have known which sample is what. We think this would have been less relevant for our study.

Did the study involve field work? ☐ Yes ☒ No

Reporting for specific materials, systems and methods

We require information from authors about some types of materials, experimental systems and methods used in many studies. Here, indicate whether each material, system or method listed is relevant to your study. If you are not sure if a list item applies to your research, read the appropriate section before selecting a response.

Materials & experimental systems

n/a	Involved in the study
☒	☐ Antibodies
☒	☐ Eukaryotic cell lines
☒	☐ Palaeontology and archaeology
☒	☐ Animals and other organisms
☒	☐ Clinical data
☒	☐ Dual use research of concern
☒	☐ Plants

Methods

n/a	Involved in the study
☒	☐ ChIP-seq
☐	☒ Flow cytometry
☒	☐ MRI-based neuroimaging

Plants

Seed stocks	N/A
Novel plant genotypes	N/A
Authentication	N/A

Flow Cytometry

Plots

Confirm that:

☒ The axis labels state the marker and fluorochrome used (e.g. CD4-FITC).

☒ The axis scales are clearly visible. Include numbers along axes only for bottom left plot of group (a 'group' is an analysis of identical markers).

☒ All plots are contour plots with outliers or pseudocolor plots.

☒ A numerical value for number of cells or percentage (with statistics) is provided.

Methodology

Sample preparation	We focus on bacterial cells that are washed from soil samples and stained with Syto-9. Inoculants are differentiated on the basis of their genetically encoded mCherry fluorescence.
Instrument	CytoFLEX Flow Cytometer (Beckman Coulter).
Software	Data are collected by the instrument, saved as fcs3-files, which were then read with fca_readfcs in Matlab (v. 2021).
Cell population abundance	Bacterial cell abundances vary between 10,000 and 1,000,000 events per sample.
Gating strategy	We define the positive cell fraction on the basis of comparison to a non-Syto9 stained control of pure inoculant cultures, and on the basis of comparison to a non-inoculant soil - extracted in the same way, to defined potential particle background. We then use log plots of FSC/SSC versus FL1H (for SYTO9) or FL2H (for mCherry), to define the gate and count particle abundances in that gate. The CytoFlex has volumetric counting and we collect the data within 10 µl volume, at slow flow rate.

☒ Tick this box to confirm that a figure exemplifying the gating strategy is provided in the Supplementary Information.
